# Comprehensive analysis of circRNAs from cashmere goat skin by next generation RNA sequencing (RNA-seq)

**DOI:** 10.1038/s41598-019-57404-9

**Published:** 2020-01-16

**Authors:** Yuanyuan Zheng, Taiyu Hui, Chang Yue, Jiaming Sun, Dan Guo, Suling Guo, Suping Guo, Bojiang Li, Zeying Wang, Wenlin Bai

**Affiliations:** 1 College of Animal Science &Veterinary Medicine, Shenyang Agricultural University, Shenyang, 110866 China; 2 Liaoning Modern Agricultural Production Base Construction Engineering Center, Liaoyang, 111000 China; 3 Prosperous Community, Changshun Town, Huade 013350 China

**Keywords:** Genetics, Zoology

## Abstract

Circular RNA (circRNA) is endogenous non-coding RNA (ncRNA) with a covalently closed circular structure. It is mainly generated through RNA alternative splicing or back-splicing. CircRNA is known in the majority of eukaryotes and very stable. However, knowledge of the circRNA involved in regulating cashmere fineness is limited. Skin samples were collected from Liaoning cashmere goats (LCG) and Inner Mongolia cashmere goats (MCG) during the anagen period. For differentially expressed circRNAs, RNA sequencing was performed, and the analysis led to an identification of 17 up-regulated circRNAs and 15 down-regulated circRNAs in LCG compared with MCG skin samples. In order to find the differentially expressed circRNAs in LCG, we carried out qPCRs on 10 candidate circRNAs in coarse type skin of LCG (CT-LCG) and fine type skin of LCG (FT-LCG). Four circRNAs: ciRNA128, circRNA6854, circRNA4154 and circRNA3620 were confirmed to be significantly differential expression in LCG. Also, a regulatory network of circRNAs-miRNAs was bioinformatically deduced and may help to understand molecular mechanisms of potential circRNA involvement in regulating cashmere fineness.

## Introduction

The goat (*Capra hircus*), is economically important livestock, used in the production of cashmere, meat, and milk. The Liaoning cashmere goat breed (LCG) is famous for high fiber production^1^, whereas Inner Mongolia cashmere goats (MCG) produce high-quality cashmere fiber compared with other cashmere goat breeds^2^. In recent years, the characteristics of cashmere fiber have received special attention in that they play an obvious role in cashmere quality. Several studies indicated that coding and noncoding genes were associated with the regulation of cashmere growth^3–5^. In addition, several important pathways have been demonstrated to be related to the formation of cashmere fiber^6–8^, for instance, Wnt, NF-κB, Shh, Notch and other signaling pathways^9–13^. However, there are no systematic studies on the molecular regulation of cashmere fineness in the skin.

Circular RNAs (CircRNAs), a new class in the eukaryotic transcriptome, are characterized by the 3′ and 5′ ends of which are covalently linked in a covalently closed loop without free ends^14,15^. CircRNAs, with the unique circular structure, are more stable and have longer half-lives than mRNAs^16,17^. More recently, many studies have demonstrated that circRNAs contribute to the generation of cancer^18,19^, regulate gene expression in many biological processes, and participate in the occurrence and development of various diseases^20^. In a study by Li *et al*. identified 6,113 circRNAs from muscles of sheep by RNA-seq^21^, and a total of 10,226 circRNAs were detected from pituitary glands of sheep by RNA-seq^22^. Zheng *et al*. revealed that circRNAs can act as a microRNA sponge to isolate microRNA by competing with targeted mRNA^23^. Another investigation determined that 151 circRNAs were differently expressed in ORFV-infected goat skin fibroblast cells and uninfected cells^24^. The mechanism of potential involvement of circRNAs in cashmere formation remains unclear.

In the present study, we aim to find differentially expressed circRNAs in cashmere goat skin. We used RNA-seq to identify the circRNAs in LCG and MCG skin samples, and hundreds of circRNAs were obtained in goat skins. To further explore the relationship between circRNAs with cashmere fineness and its potential role, we also generated a regulatory network that took into account interactions between these circRNAs and miRNAs. Our findings may offer a new insight into cashmere goat circRNAs and their potential involvement in regulation of cashmere fineness.

## Results

### Identification of circRNAs in cashmere goat skin

**Figure 1:**
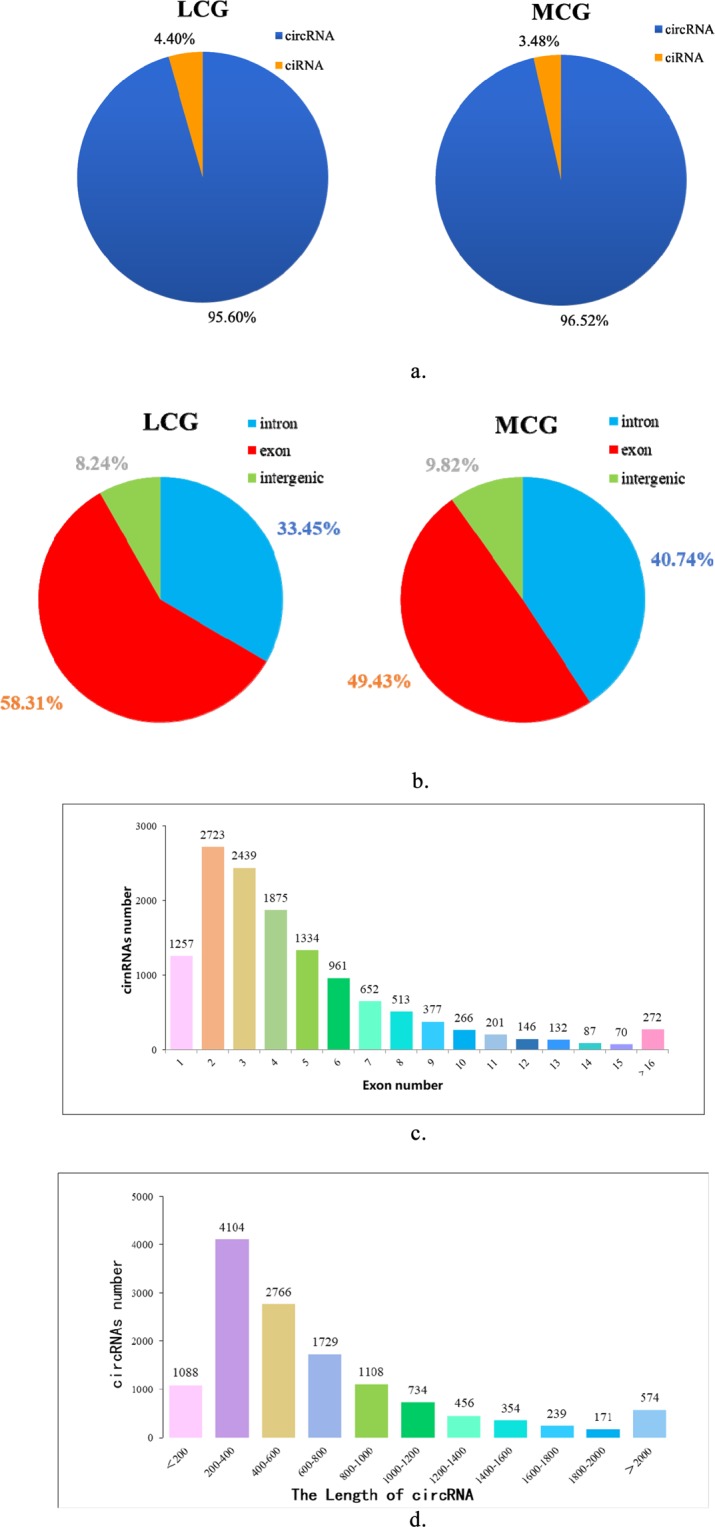
Information on circRNAs from RNA-seq in Liaoning cashmere goats (LCG) and Inner Mongolia cashmere goats (MCG) skin tissue. (**a**) The types of circRNAs. (**b**) Distribution of exons, introns, and intergenic circRNAs. (**c**) The number of exons in circRNAs. (**d**) The lengths of circRNAs.

In order to understand the differentially expressed circular RNAs in goat skin, we performed RNA-seq analysis processes in LCG and MCG skin. Total clean reads were obtained after deleting the low-quality raw reads, the mapping ratios of clean reads were 91.27% and 84.93% in LCG and MCG. A total of 13,320 circRNAs were identified from the RNA-seq data, including 610 circular intronic RNAs (ciRNAs) (Fig. 1a**)**. There are 7,531 and 8,943 circRNAs detected in LCG and MCG libraries, respectively. The MCG samples (49.43%) were compared with the LCG samples (58.31%), and the percentage of mapped sequence reads that could be aligned with the exon region were significantly lower (Fig. 1b). The lengths of circRNAs ranged from 200 to 400 bp in LCG and MCG (Fig. 1c), and the majority of circRNAs contained 2–7 exons (Fig. 1d). We found that the genomic loci, from which the circRNAs were derived, were over 29 autosomes and X chromosomes in the two types of samples.

### Differentially expressed circRNAs in LCG and MCG

**Figure 2:**
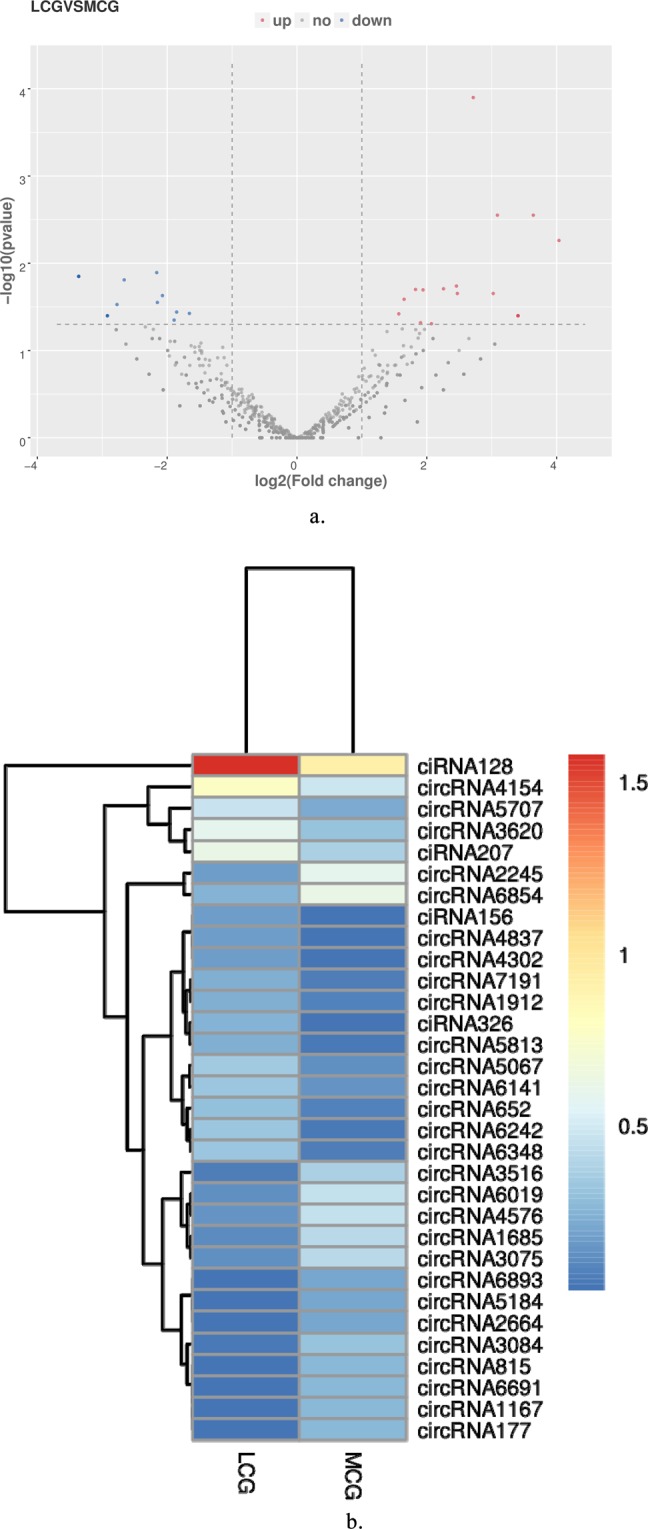
Differentially expressed circRNAs in LCG and MCG. (**a**) Volcano map of differentially expressed circRNAs. Red dots indicate up-regulation and blue dots indicate down-regulation. (**b**) Cluster heatmap of differentially expressed circRNAs. The sample is represented by the abscissa and the log value of circRNA expression is regarded by the ordinate, which means that the heatmap is drawn from log10 of circRNA expression. The highly expressed circRNA is indicated by red, meanwhile, the lowly expressed circRNA is presented by blue.

**Figure 3:**
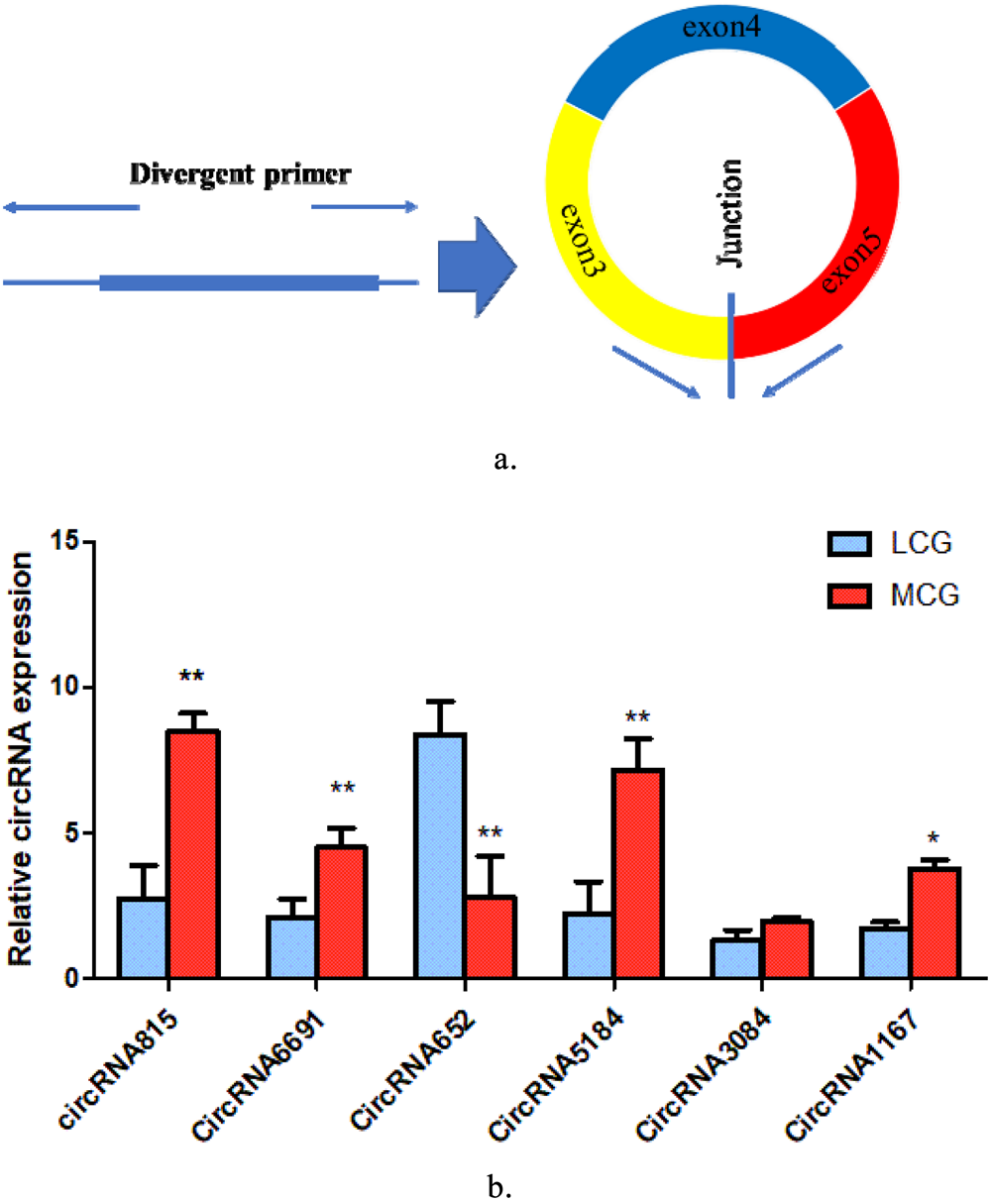
Quantitative real-time PCR (qPCR) result of circRNAs expression. (**a**) Divergent primers used in the amplification of circular junctions. (**b**) Validation of putative circRNAs by qPCR. Blue: LCG; Red: MCG. Error bars indicate mean ± SE for three individuals, “*”*p* < 0.05, “**”*p* < 0.01.

A total of 32 circRNAs were identified as differentially expressed when we compared the data between LCG and MCG skin tissues (Fig. 2a), of which 17 circRNAs were significantly up-regulated and 15 circRNAs were significantly down-regulated in the LCG (Tables 1 and 2**)**. Then, we used a cluster heat-map analysis of differentially expressed circRNAs to better understand their potential relationship (Fig. 2b). To assure the accuracy of RNA-seq strategy, six differentially expressed circRNAs were randomly selected and specific qPCR primers were designed within the circRNAs’ junction regions (Fig. 3a). The expression levels of circRNAs determined by qPCR and RNA-seq are highly consistent (Fig. 3b). This meaning the significant reliability of RNA-seq data acquisition and subsequent analysis procedures in this study.

**Table 1:** Up-regulated circRNAs in LCG and MCG.

circRNA ID	host gene	LCG (*FPKM*)	MCG (*FPKM*)	log2FC	*p*-value
*ciRNA128*	*TCHH*	36.95	7.14	2.72	0.00012611
*circRNA4154*	*HOMER3*	5.05	2.04	1.65	0.02588325
*ciRNA207*	*HCFC1R1*	3.14	1.34	1.57	0.03802090
*circRNA3620*	*CAMSAP1*	2.86	1.02	1.83	0.01997006
*circRNA5707*	*CREB5*	1.97	0.64	1.94	0.02020643
*circRNA5067*	*TMEM62*	1.24	0.32	2.26	0.01961495
*circRNA6242*	*SPTBN4*	1.12	0.11	3.64	0.00281198
*circRNA6348*	*ARID1A*	1.12	0.16	3.09	0.00281198
*circRNA6141*	*GSE1*	1.12	0.38	1.90	0.04803681
*circRNA652*	*CDC6*	0.95	0.21	2.46	0.01825840
*ciRNA326*	*PDLIM2*	0.79	0.05	4.04	0.00547933
*circRNA7191*	*PHLPP1*	0.73	0.16	2.47	0.02220809
*circRNA5813*	*ISPD*	0.73	0.11	3.02	0.02220809
*circRNA1912*	*RYK*	0.73	0.21	2.07	0.04933581
*circRNA4837*	*CASC4*	0.51	0.05	3.40	0.03997049
*circRNA4302*	*GFM2*	0.51	0.05	3.40	0.03997049
*ciRNA156*	*GRHL1*	0.51	0.05	3.40	0.03997049

**Table 2:** Down-regulated circRNAs in LCG and MCG.

circRNA ID	host gene	LCG (*FPKM*)	MCG (*FPKM*)	log2FC	*p*-value
*circRNA6854*	*KCTD9*	0.79	3.17	-1.66	0.03757668
*circRNA2245*	*HEBP1*	0.51	2.90	-2.16	0.01279541
*circRNA4576*	*MED17*	0.39	1.82	-1.85	0.03625281
*circRNA6019*	*AXDND1*	0.34	1.82	-2.07	0.02348295
*circRNA3075*	*PRPF18*	0.34	1.61	-1.89	0.04479169
*circRNA1685*	*PHLDB2*	0.28	1.61	-2.15	0.02812314
*circRNA3516*	*UBXN2A*	0.17	1.39	-2.66	0.01551409
*circRNA3084*	*FAM188A*	0.11	1.02	-2.77	0.02972176
*circRNA1167*	*REV3L*	0.06	0.80	-3.36	0.01414588
*circRNA177*	*BMS1*	0.06	0.80	−3.36	0.01414588
*circRNA6691*	*PHLDB2*	0.06	0.80	−3.36	0.01414588
*circRNA815*	*ARL8B*	0.06	0.80	−3.36	0.01414588
*circRNA5184*	*AP3B1*	0.06	0.59	−2.92	0.03997049
*circRNA2664*	*VPS72*	0.06	0.59	−2.92	0.03997049
*circRNA6893*	*AGTPBP1*	0.06	0.59	−2.92	0.03997049

**Figure 4:**
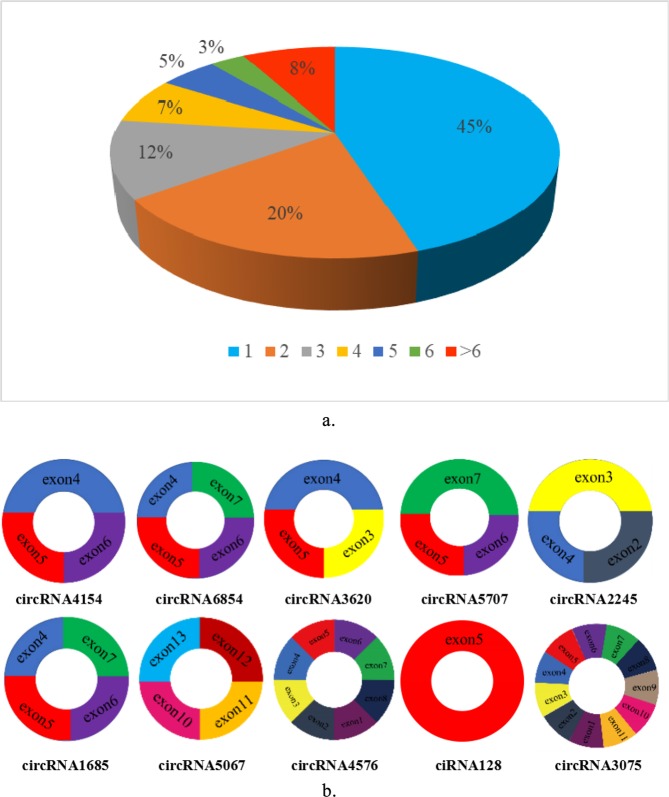
(**a**) Numbers of circRNAs produced by the same gene. (**b**) Exon distribution of candidate circRNAs. Different colors represent different exons.

In our data, total 13,320 circRNAs detected in our study were derived from 4826 host genes. 45% of these host genes generated only one circRNA, and 20% of these host genes generated two circRNAs, whereas 8% of these host genes generated more than six circRNAs (Fig. 4a). We screened 10 candidate circRNAs and found these circRNAs consisted of three or four exons on average (Fig. 4b).

### Enrichment analysis of differentially expressed circRNA host genes

**Figure 5:**
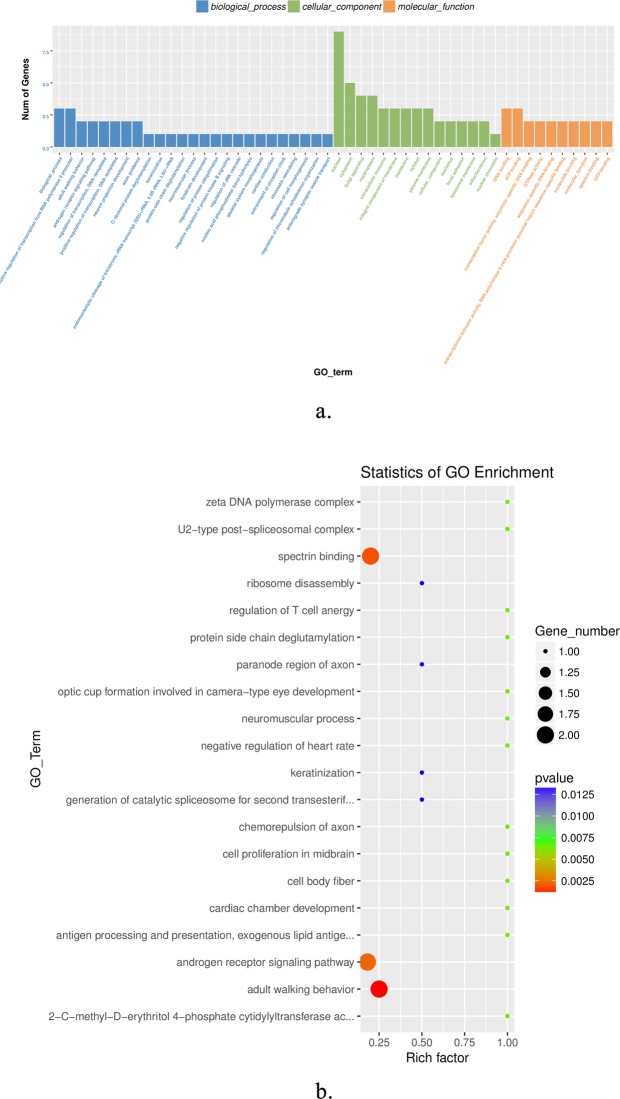
Gene ontology (GO) analysis of differentially expressed circRNAs. (**a**) Top 25 biological processes, top 15 cell components, and top 10 molecular functions. (**b**) The top 20 GO terms. The color of the dot corresponds to different *p-value* ranges, and the size of the dot indicates the number of genes in the pathway. Rich factor denotes the number of differentially expressed circRNAs in the GO/ the total number of circRNAs in the GO.

**Figure 6:**
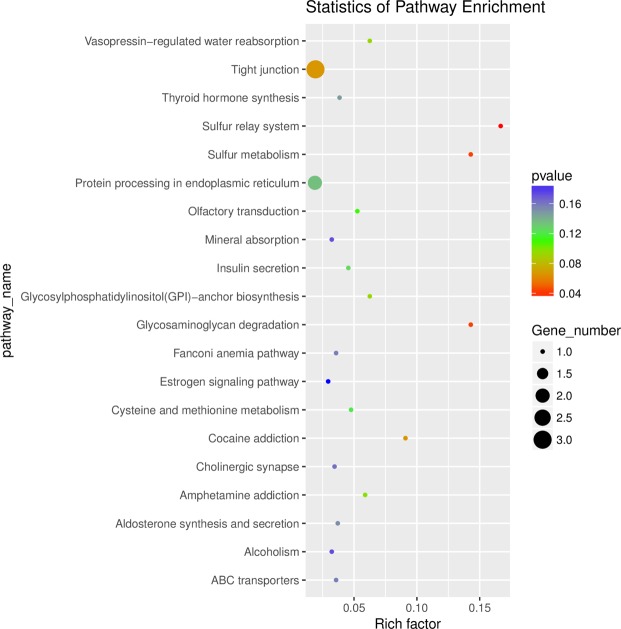
Top 20 Kyoto Encyclopedia of Genes and Genomes (KEGG) pathways of host genes of differentially expressed circRNAs. The color of the dot corresponds to different *p-value* ranges, and the size of the dot indicates the number of genes in the pathway. Rich factor denotes the number of differentially expressed circRNAs in the KEGG/the total number of circRNAs in the KEGG.

We performed GO and KEGG enrichment analysis for the host genes of differentially expressed circRNAs. A total of 22 host genes were enriched in 106 GO terms, and the top 25, top 15 and top 10 in biological processes, cellular components and molecular functions, respectively (Fig. 5a). In Fig. 5b, the top 20 significant GO terms were exhibited, and it can be seen that keratinization and intermediate filament organization were closely related to cashmere fiber growth. A total of 43 pathways were enriched using KEGG analysis, and the top 20 pathways were shown in Fig. 6. These include the sulfur relay system, sulfur metabolism, and glycosaminoglycan degradation pathways, suggesting that these pathways could also be involved in the regulation of cashmere fineness.

### Analysis of interactions between circRNAs and miRNAs

**Figure 7:**
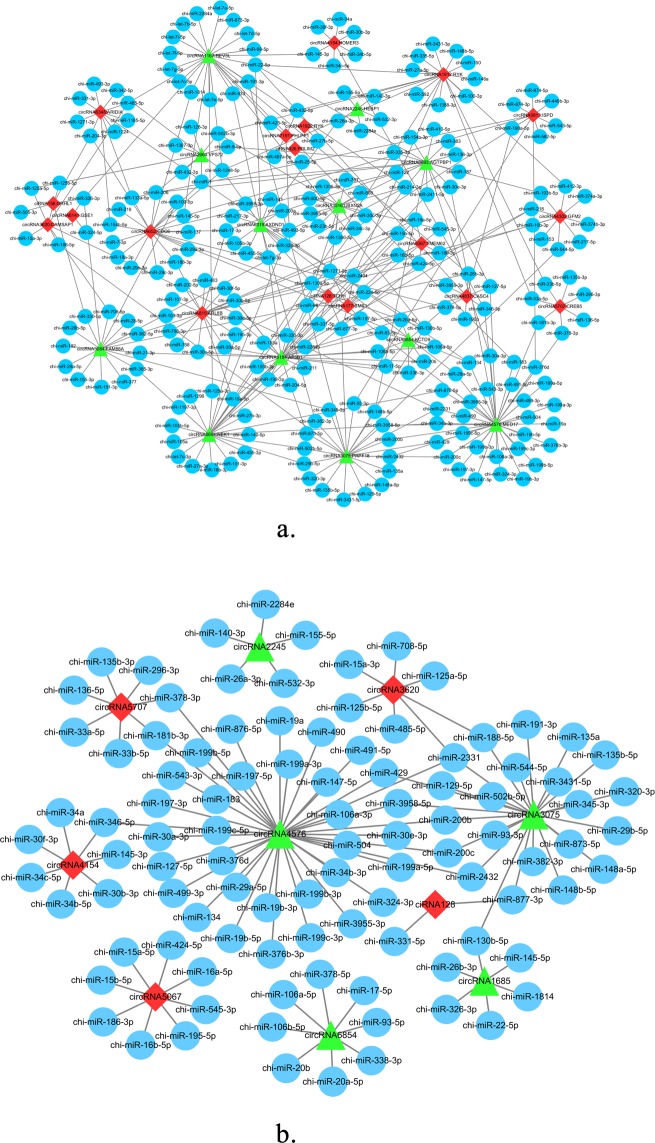
The circRNAs-miRNAs network. (**a**) Network of 32 differentially expressed circRNAs. Red and green represent up- and down-regulation, and blue represents target-miRNA. (**b**) The circRNAs-miRNAs network of 10 candidate circRNAs.

It is generally accepted that circRNA is an adsorbed miRNA sponge and interacts with miRNA. We predicted the potential circRNAs-miRNAs interactions for these differential circRNAs, and the results indicated that the co-expression networks included 32 differentially expressed circRNAs, their host genes and 244 miRNAs (Fig. 7a). The results suggested that circRNA6854 may function as a sponge for these miRNAs, such as chi-miR-106a-5p, chi-miR-106b-5p, chi-miR-17-5p, chi-miR-20a-5p, chi-miR-20b, chi-miR-338-3p, chi-miR-378-5p, and chi-miR-93-5p. CiRNA128 has an interaction with chi-miR-331-5p and chi-miR-877-3p. Moreover, the interactions between 10 identified differentially expressed candidate circRNAs and their target miRNAs are presented in Fig. 7b.

### Validation of differentially expressed circRNAs by qPCR

**Figure 8:**
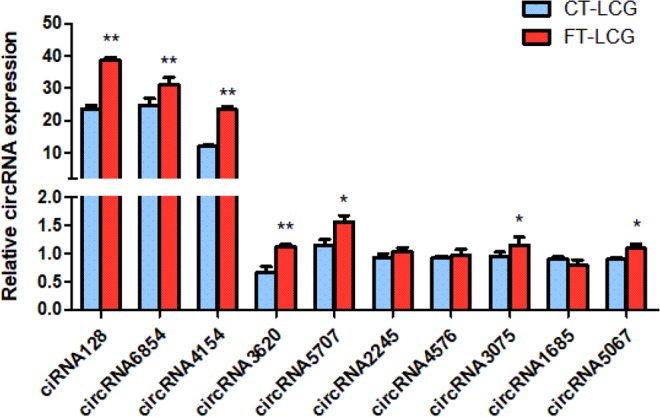
Quantitative real-time PCR results of circRNAs expression. Blue: CT-LCG; Red: FT-LCG. Error bars represents standard deviations within the group, the “*”indicates the significant difference *p* < 0.05, “**”indicates *p* < 0.01.

To investigate the expression of circRNAs and determine circRNAs may be vital for regulating cashmere fineness in LCG skin tissue, we used qPCR to confirm the differential expression of certain circRNAs in coarse type and fine type LCG skins. Ten differentially expressed circRNAs were selected and specific qPCR primers were designed within the circRNAs’ junction regions. RNA-seq results showed that ciRNA128 had the highest expression level among the up-regulated circRNAs, while circRNA6854 had the highest expression level among the down-regulated circRNAs. The qPCR experiment results of CT-LCG and FT-LCG were shown in Fig. 8. It was proven that these circRNAs really existed and showed similar expression patterns in LCG skin, with the majority exhibiting a higher expression level in FT-LCG. The results of ciRNA128, circRNA6854, circRNA3620 and circRNA4154 are significantly differential expressed in RNA-seq and qPCR, which suggests that they might play a positive role in cashmere goats with different fiber diameters.

## Discussion

The cashmere goat is a great breed that produces large amounts of high-grade cashmere fiber. As one of the largest producers of cashmere in the world, China has made tremendous contributions to the world animal fiber industry and plays an indispensable role in global cashmere production^25^. CircRNAs can be classified into four categories: ecRNA, EIciRNA, ciRNA, and tricRNA^26^. CircRNAs located in the nucleus are mainly involved in transcriptional regulation. During the past few years, through high-throughput RNA sequencing and bioinformatics analysis, a great number of circRNAs have been discovered in different species and tissues. For example, 13,950 circRNAs were detected in pre-ovulatory ovarian follicles of goats, and 37 circRNAs were found to be differentially expressed^27^. Empirical Bayes sequencing analysis identified 11 down-regulated and 32 up-regulated circRNAs in embryos with black fur skin and white fur skin of mice, and these circRNAs may play a role in skin pigmentation^28^. The effects of mRNA and lncRNA have been reported on the skin and hair follicles^29,30^, but there are few studies on the effect of circRNAs on the fineness of cashmere and cashmere growth.

In recent years, numerous studies have found that circRNAs located in cytoplasm can compete with mRNAs for target binding sites of miRNAs to regulate the expression of mRNAs. The interaction between circRNA and miRNA has attracted more and more attention. In fact, a number of non-coding RNAs have been identified and reported in cashmere goat skin^31–33^. In the current study, we identified a total of 13,320 circRNAs in goat skins using RNA-seq analysis. Among these circRNAs, 32 circRNAs were differentially expressed between LCG and MCG skin, and then we randomly selected 6 circRNAs to verify the expression levels by qRT-PCR. The results of RNA-seq and qPCR were almost identical, thereby indicated the reliability of RNA-seq. Additionally we carried out validation on 10 circRNAs in CT- LCG and FT- LCG, interestingly, these circRNAs also had a high expression level in FT-LCG, which may play potential positive role in regulating fiber fineness formation.

We obtained 106 terms from GO enrichment analysis, including 69 biological processes, 17 molecular functions, and 20 cellular components. Keratinization, intermediate filament organization, spindle midzone, Wnt-protein binding, and Wnt-activated receptor activity negative regulation of stress fiber assembly were significant enriched, these pathways may participate in the regulation of cashmere fineness formation. Although there were only three pathways in which the sulfur relay system, sulfur metabolism, and glycosaminoglycan degradation were found to be significantly enriched, the host genes of circRNA6854 and circRNA815 were involved in all pathways. Studies have shown that intermediate filaments are probably the key factors involved in cashmere growth^34^. In addition, several important pathways that play a role in dominating hair follicle development were reported, such as the PPAR pathway^35^, Wnt signaling pathway^5,36,37^, MAPK signaling pathway^38^, and NF-kappa B signaling pathway^39^. Our data also enriched these pathways, it further illustrates the importance of these circRNAs in goat skin.

Previous studies have shown that circRNA can further influence the expression of target miRNA by acting as a miRNA sponge^40–42^, and the interactions between circRNA and miRNA have been investigated^16,43^. It is noteworthy that some known miRNAs have been reported to be closely related to cashmere growth and development, while some miRNAs may play multiple roles in cashmere goat skin in the growth period. The expressions of mir-103-3p, miR-15b-5p, miR-17-5p, mir-30c-5p, mir-200b, mir-199a-3p, mir-199a-5p, mir-30a-5p, and mir-29a-3p were significantly different between anagen and telogen skin in Liaoning Cashmere goats^44^, and these were found as the target miRNAs of circRNAs in our data. We hypothesized that a lot of circRNAs interact with many cashmere-related miRNAs (chi-miR-106a-5p, chi-miR-106b-5p, chi-miR-17-5p, let-7b-5p, chi-miR-20b, chi-miR-143). Previous research proved that oar-miR-103-3P, oar-miR-148b-3P, oar-miR-320-3P, oar-miR-31-5P, oar-novel-1-5P, and oar-novel-2-3P may play an important role in follicle growth of Tibetan Sheep^45^. Mir-200b as a target was involved in the regulation of hair follicle development^46^, while miR-1839, miR-374b, and miR-2284n have been reported as showing the highest relative expression levels at the anagen in Inner Mongolia cashmere goat skin tissue^33^. It was reported that let-7b-5p, mir-10a-5p, and mir-21-5p exhibited differences at various hair cycle stages in mouse skin^47^. Research showed that the gene families let-7, mir-17, mir-30, mir-15, and mir-8 were highly expressed in goat skin^31^. In Hu sheep lambskin hair follicles, 14 miRNAs including miR-143, miR-10a, and let-7 were screened as important candidate miRNAs^48^. MiR-143, miR-203, and let-7, let-7b, let-7b-5p, let-7f, and let-7c were found to be expressed in Liaoning Cashmere Goats and Fine-Wool Sheep skin^49^, and the let-7 family was reported to be involved in the regulation of cell differentiation^50^.  It suggests that let-7b-5p may affect cashmere development as the target of circRNA1167. MiR-378, miR-378e, and miR-378d were only detected in Liaoning Cashmere Goats and promoted angiogenesis^50,51^. Five novel miRNAs (chi-miR-2284n, chi-miR-421*, chi-miR-421, chi-miR-1839, and chi-miR-374) play roles in the production of cashmere in Inner Mongolia cashmere goat skin^33^. Taken together, it can be inferred that ciRNA128-chi-miR-331-5p and circRNA6854-chi-miR-17-5p may have certain roles in cashmere fineness and cashmere fiber morphogenesis.

The expression of circRNAs has been appropriately correlated with an abundance of host genes in different animal tissues^52–55^, such as oar_circ_0003451 and TTN, and oar_circ_0005250 and MYH7 may play important roles in muscle development and growth^22^; circRNA8077 and CRIM1, as well as circRNA3314 and TMEM159 play vital roles in the development of the receptive endometrium^56^. The host genes of circRNAs are involved in regulating hair traits, and these circRNAs may be considered as a possible factor regulating cashmere fineness. The host gene TCHH of ciRNA128 has been confirmed to be involved in hair formation^57^. Studies based on GWAs found TCHH in Latin Americans of mixed European and Native American origin^58^. Among Europeans, the strongest link between straight hair and TCHH was found^59^, and DSC2, DSG3, CALML5, TCHH are related to hair growth using iTRAQ-labeling in sheep and goats^60^. KCTD9 has been reported to be associated with cancer^61^, promoting cell growth and inhibiting cell activation^62,63^. Thus, it proved potential reference value for cashmere fiber fineness and the expression analysis of circRNAs in LCG.

In conclusion, we performed RNA-seq analysis that identified 13,320 circRNAs in cashmere goat skins, of which 32 circRNAs were found to be differential expression. The result of qRT-PCR confirmed that four circRNAs (ciRNA128, circRNA6854, circRNA4154 and circRNA3620) were differentially expressed in CT-LCG and FT-LCG. Host genes of differentially expressed circRNAs were mainly enriched in keratinization and intermediate filament organization. An integrated regulatory network of circRNAs and miRNAs was executed in anagen cashmere goat skin. This study may contribute to better understanding of circRNAs in goat skin.

## Materials and Methods

### Ethics statement

All experiments in this study were approved and conducted according to the Animal Experimental Committee of Shenyang Agricultural University, Shenyang, China (201606005).

### Sample preparation

Skin samples from three adult female Liaoning cashmere goats (*d* = 19.4 µm, 19.5 µm and 19.8 µm) and three adult female Inner Mongolia cashmere goats (*d* = 13.8 µm, 14.0 µm and 14.1 µm) were carefully collected. The animals we collected were based on all the same conditions, including sex, age, feeding and physiological status and other factors. To reduce pain to experimental animals, we used local anesthesia with procaine. In the upper one-third of the right scapula along the mid-dorsal and mid-abdominal lines, about 1 cm^2^ lateral skin from the six cashmere goats were taken and disinfected with 75% ethanol. And then, the skin samples were washed three times with PBS and immediately stored in liquid nitrogen until RNA isolation. In addition, three coarse type (CT) skin (*d* = 19.5 µm, 19.7 µm and 20.2 µm) and three fine type (FT) skin (*d* = 15.3 µm, 15.4 µm and 15.6 µm) samples from Liaoning cashmere goats were obtained with the same method for qRT-PCR analysis.

### Total RNA isolation, library construction and sequencing

The total RNA amount and purity of each sample was quantified by Nano Drop ND-1000 (Nano Drop, Wilmington, DE, USA). Approximately 5 ug of total RNA was used to deplete ribosomal RNA according to the manufacturer’s instructions for the Ribo-Zero rRNA Removal Kit (Illumina, San Diego, USA). In order to construct the cDNA library of circRNAs, we used Rnase R to remove linear RNA. The average insert size for the final cDNA library was 300 bp (±50 bp), the library was purified and qualified by Agilent Bioanalyzer 2100 system.

### Identification of circRNAs and analysis of differentially expressed circRNAs

The cDNA libraries were performed the paired-end sequencing on an Illumina Hiseq. 4000 (LC Bio, China) following the vendor’s recommended protocol. Firstly, low-quality reads and adapters were removed by Cutadapt v1.10, quality controlled by FastQC v0.10.1, and then obtained the high-quality clean reads. TopHat v2.0.4 was utilized to map the clean reads to the reference genome from National Center for Biotechnology Information (NCBI) (https://www.ncbi.nlm.nih.gov/genome/?term = Capra + hircus)^64,65^. Also, StringTie v1.3.0 was used to assemble and quantify expressed genes and transcripts (https://ccb.jhu.edu/software/stringtie/index.shtml)^66^. CIRCExplorer2 v2.2.6 software and the following criteria were used to identify candidate circRNAs: mismatch ≤2, back-spliced junction reads ≥1, and distances of two splice sites of less than 100 kb in the genome^67^. Then, the back-spliced reads with at least two supporting reads were annotated as circRNAs. The differential expression of circRNAs between the two groups was assessed using the Ballgown package. A *p*-value < 0.05 and |log2 (fc)| > 1 were set as the threshold for differential expression^68,69^.

### Gene ontology (GO) analysis and KEGG analysis of host genes

GO analysis (http://www.geneontology.org) was applied to differentially expressed circRNA-hosting genes. Similarly, pathway analysis uncovered the significant pathways related to differentially expressed circRNAs according to the annotation of the Kyoto Encyclopedia of Genes and Genomes (KEGG) (http://www.kegg.jp/kegg)^70^. A threshold of *p* < 0.05 was used as a criterion for the determination of whether the enrichment analysis was significant^71^.

### Network construction of the circRNAs-miRNAs interaction

The interaction of circRNAs-miRNAs was predicted with miRNA target prediction software miRanda (http://www.microrna.org/microrna/home.do) and TargetScan (http://www.targetscan.org/)^72^, where the max free energy values of miRanda is <−10 and the score percentiles of TargetScan is ≥50. The differential expression circRNAs-miRNAs interaction and the network of circRNAs along with their target miRNAs were performed using cytoscape v3.5.1 software (https://cytoscape.org/, USA)^73^.

### Quantitative real-time PCR validation

We randomly detected 6 differentially expressed circRNAs for qRT-PCR. To prove the resistance of circRNAs to RNase R digestion, we treated total RNAs with RNase R before cDNA synthesis. In order to validate the differentially expressed circRNAs, total RNAs were synthesized directly to cDNA synthesis by an RT-PCR kit. According to the manufacturer’s instructions, Real-time PCR was performed using SYBR Green (TaKaRa Biotech, Dalian). The glyceraldehyde-3-phosphate dehydrogenase (GAPDH) gene was used as an internal control to normalize the expression level of circRNAs^74^. Three independent experiments were carried out on LCG and MCG skin samples. Six pair primers were designed by primer 5 software (www.premierbiosoft.com) and listed in Supplemental Table S1, and all primers were spanning the distal ends of circRNAs. The relative expression levels of different circRNAs were analyzed by the 2^*−*ΔΔCt^ method in qPCR data^75^. The data were indicated as the means ± SE (n = 3). All statistical analyses in the two groups were calculated using a t-test in SPSS statistical software (Version 22.0, Chicago, IL, USA), the difference was significant at *p* < 0.05. In addition, three CT-LCG and FT-LCG skin samples were verified by qPCR under the same experimental conditions to find the differential circRNAs in LCG.

## Electronic supplementary material

Supplementary Information.
