# Supplementary material for: Comprehensive analysis of circRNAs from cashmere goat skin by next generation RNA sequencing (RNA-seq)
Source: Sci Rep. 2020 Jan 16;10:516. doi: 10.1038/s41598-019-57404-9 (PMC6965140; doi:10.1038/s41598-019-57404-9)
Supplement: Supplementary file 1 — Supplementary Information. [file 41598_2019_57404_MOESM1_ESM.pdf]

Comprehensive analysis of circRNAs from cashmere goat skin by next generation RNA sequencing (RNA-seq)

Yuanyuan Zheng[1], Taiyu Hui[1], Chang Yue[1], Jiaming Sun[1], Dan Guo[2], Suling Guo[3], Suping Guo[1], Bojiang Li[1], Zeying Wang[1,*], Wenlin Bai[1,*]

Table S1. Primer of circRNAs

name	Primer sequence	Annealing temperature (℃)	product
C-GAPDH-F:	5' TGTTTGTGATGGGCGTGAA 3'	58.4	173bp
C-GAPDH-R:	5' AGTCTTCTGGGTGGCAGTGAT 3'	58.4	
circRNA815-F	5' CTAATGCCTTGGATGAGAAACA 3'	57.4	155bp
circRNA815-R	5' ATGTTGAAGCCCACTGTAGGTAT 3'	57.6	
ciRNA6691-F	5' GGAGCACAACCTCTACCACCTA 3'	58.0	214bp
ciRNA6691-R	5' CCTCCTTCACAGTAATCCATCAC 3'	58.6	
ciRNA652-F	5' ACCATATGACTGCAGAGAAGGG 3'	58.4	178bp
ciRNA652-R	5' CTGTGACTGGGAACGGGTT 3'	57.6	
circRNA5184-F	5' ACAGCCTTGATCCAGAGCAGA 3'	59.7	146bp
circRNA5184-R	5' CCAACAATCCGTTTCATAGCAT 3'	59.5	
circRNA3084-F	5' CTCACCTCACCGTATTTTTTGC 3'	59.3	183bp
circRNA3084-R	5' ATGCCTGAACAGGTGCAATAAC 3'	59.4	
circRNA1167-F	5' TGGGAAGAAGGCGAAATACC 3'	59.3	204bp
circRNA1167-R	5' TGGCACATAGAGGTAAGGAAAGA 3'	58.3	
